# Relationship between COVID-19 Cases and Environmental Contaminants in Quito, Ecuador

**DOI:** 10.3390/ijerph21101336

**Published:** 2024-10-09

**Authors:** Andrea Damaris Hernández-Allauca, Carlos Gabriel Pérez Castillo, Juan Federico Villacis Uvidia, Paula Abdo-Peralta, Catherine Frey, Guicela Margoth Ati-Cutiupala, Juan Ureña-Moreno, Theofilos Toulkeridis

**Affiliations:** 1 Faculty of Natural Resources, Escuela Superior Politecnica de Chimborazo, Panamericana Sur, km 1 ½, Riobamba EC-060155, Ecuador; paula.abdo@espoch.edu.ec (P.A.-P.); guicela.ati@espoch.edu.ec (G.M.A.-C.); 2 Independent Researcher, Riobamba EC-060155, Ecuador; calin86gpec@yahoo.es (C.G.P.C.); catherine.frey@espoch.edu.ec (C.F.); kiko15821@hotmail.com (J.U.-M.); 3 Faculty of Accounting and Auditing, Technical University of Ambato, Ambato EC-180207, Ecuador; jf.villacis@uta.edu.ec; 4 School of Geology, Aristotle University of Thessaloniki, 54124 Thessaloniki, Greece; theousfq@yahoo.com

**Keywords:** infectious diseases, COVID-19, atmospheric contaminants, statistical tests, linear regression

## Abstract

The relationship between COVID-19 infections and environmental contaminants provides insight into how environmental factors can influence the spread of infectious diseases. By integrating epidemiological and environmental variables into a mathematical framework, the interaction between virus spread and the environment can be determined. The aim of this study was to evaluate the impact of atmospheric contaminants on the increase in COVID-19 infections in the city of Quito through the application of statistical tests. The data on infections and deaths allowed to identify the periods of greatest contagion and their relationship with the contaminants O_3_, SO_2_, CO, PM_2.5_, and PM_10_. A validated database was used, and statistical analysis was applied through five models based on simple linear regression. The models showed a significant relationship between SO_2_ and the increase in infections. In addition, a moderate correlation was shown with PM_2.5_, O_3_, and CO, and a low relationship was shown for PM_10_. These findings highlight the importance of having policies that guarantee air quality as a key factor in maintaining people’s health and preventing the proliferation of viral and infectious diseases.

## 1. Introduction

The coronavirus (COVID-19) pandemic has marked a turning point in contemporary history. Over time, various outbreaks of viral epidemic diseases have emerged [1]. However, COVID-19 stands out as one of the most tragic challenges in a century, after World War II [2]. This disease is caused by severe acute respiratory syndrome coronavirus 2 (SARS-CoV-2) [3,4], which presents symptoms such as fever, dry cough, and difficulty breathing [5]. Since the first case was identified in Wuhan, China, in December 2019 [6], the virus has spread rapidly throughout the world [7,8], triggering an unprecedented impact at a social, economic, and environmental level [9,10].

Initially, several studies reported that COVID-19 had a low mortality rate (2.3%) [11,12] compared to MERS (34.4%) and SARS (9.2%) [13]. However, a research study found that the number of patients with COVID-19 doubled every 6.4 days, indicating that it was a more dangerous and infectious disease than expected [14]. This spread generated a global crisis [9] that challenged health systems [15] and highlighted the vulnerability of populations to emerging diseases.

The scientific community has been interested in understanding the spread of the virus and its determining factors [16,17]. According to several studies, environmental factors can affect the epidemiological dynamics of many infectious diseases [11,18]. Among these factors, special attention has been paid to the possible influence of air quality on the incidence of respiratory diseases such as COVID-19 [19], considering that there is evidence indicating that air pollution can facilitate the transmission and pathogenesis of various bacterial and viral infections [20]. This pollution is the result of the presence of harmful substances in the atmosphere known as air contaminants [21], which come from various anthropogenic and natural sources [22]. Among the main contaminants are sulfur dioxide (SO_2_) [23,24], nitrogen dioxide (NO_2_), carbon monoxide (CO) [25], and ozone (O_3_) [26], in addition to fine particles (PM_10_) and ultrafine particles (PM_2.5_) [27]. This type of contamination exerts an adverse effect on human health [28] because it increases the host’s susceptibility to respiratory viral infections [29].

Several studies have suggested a correlation between exposure to air contaminants and the increase in COVID-19 cases [16,30,31,32], as well as its impact on the severity of the health of people infected by the virus [33,34]. Air contaminants such as NO_2_, O_3_, PM_2.5_, and PM_10_ can weaken the respiratory system [35]. People exposed to high levels of these contaminants may have inadequate lung function and a reduced immune response [36], especially in densely populated urban areas [37]. These areas have a higher number of viral infections and deaths due to high levels of suspended particles or ozone [38,39]. These findings are supported by research studies in countries such as China [25,31,33], the United States of America [25,40,41], and some European countries [42,43], where significant associations have been found between air contaminants and transmission or mortality from COVID-19. Furthermore, in the Latin American context, emissions of PM_10_, NO_2_, O_3,_ and CO are the main determining factors of the increase in virus infections [44]. This can be attributed to relentless population growth and urbanization, which have worsened air quality conditions in many cities in developing countries [37]. 

Ecuador has not been the exception. For instance, cities such as Quito, Guayaquil, and Cuenca with a high number of inhabitants face challenges in terms of air pollution [37,45,46,47,48]. According to the last official census carried out by the National Institute of Statistics and Censuses (INEC), Quito and Guayaquil are the cantons with the highest number of inhabitants in 2022, with 2,679,722 and 2,746,403 inhabitants, respectively [49]. Therefore, there are stations to measure the presence and incidence of contaminants [50]. These measurements are essential to evaluate air quality and its possible impact on the health of the population [51]. Therefore, this type of contamination may be an important factor to consider in the transmission and severity of COVID-19 in urban areas [52]. In this context, mathematical modeling emerges as a tool to understand the spread of infectious diseases [53]. Mathematical models are based on differential equations that describe the dynamics of the disease in a population over time [54], considering variables such as the transmission rate, the recovery rate, and the susceptibility of the population [55]. Additionally, they allow us to analyze the relationship between COVID-19 infections and atmospheric contaminants with greater precision [56] by integrating epidemiological and environmental data with sophisticated mathematical methods [57]. This may involve the use of linear regression techniques, nonlinear regression, or time series models, depending on the nature of the data and the expected relationship [58]. 

The aim of this research was to evaluate the impact of air contaminants on the increase in COVID-19 infections in the city of Quito through the application of statistical tests. For this purpose, the existence of a significant relationship between air pollution and the number of COVID-19 cases was identified. Mathematical models made it possible to establish this link through the estimates of coefficients, standard errors, and *p* values for each variable. This provided a quantitative understanding of the relationship between air quality and the spread of the virus, in which an adverse influence of atmospheric contaminants is denoted, depending on the infection cases caused by the virus.

## 2. Materials and Methods

### 2.1. Study Area

**Figure 1:**
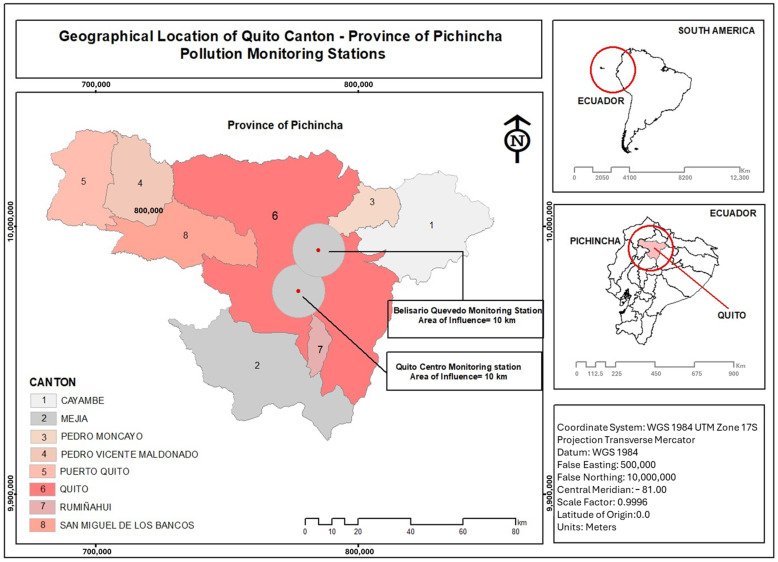
Map of study area.

This study was carried out in Quito city, province of Pichincha, Ecuador (Figure 1). Quito canton is one of the 221 cantons and is in the north center of the Andean natural region. It has an area of 372.4 km^2^ and a total of 52 parishes, 32 urban and 33 rural [59]. It has 2,679,722 inhabitants in total, 1,763,275 in the urban area and 916,447 in the rural area [49].

### 2.2. Methodology

The approach of this research was cross-sectional. The methodology applied in this study considered a quantitative research approach with an exploratory, analytical, and descriptive design. Three stages were considered: (i) COVID-19 cases; (ii) atmospheric contaminants vs. COVID-19 infections; and (iii) development of statistical tests. This was conducted through data collection, statistical data analysis, and the application of mathematical modeling.

#### 2.2.1. COVID-19 Infections

A bibliographic review was carried out to understand previous scientific evidence and obtain data necessary for the analysis of the variables involved (COVID-19 infections and air contaminants). Data on deaths for the years 2019, 2020, and 2021 were collected from the database of the National Institute of Statistics and Censuses (INEC) [60]. In addition, the data on COVID-19 infections were obtained from the database of the Ministry of Public Health of Ecuador for 2020 and 2021 [61]. 

Microsoft Excel software (version 2020) was used for data analysis and the generation of tables and figures at the national level. Total deaths in Ecuador during the year 2019 were compared with deaths during 2020 and 2021. This was intended to obtain data that corroborates the increase in deaths in the years that COVID-19 appeared. Subsequently, at the provincial level, COVID-19 deaths were compared between 2020 and 2021 in one of the four regions of the country: the Andean natural region. This region has eleven provinces: Azuay, Bolívar, Cañar, Carchi, Cotopaxi, Chimborazo, Imbabura, Loja, Pichincha, Tungurahua, and Santo Domingo of Tsáchilas [24]. These data allowed us to corroborate the presence of a greater number of infections in the province of Pichincha. Based on these data, the COVID-19 cases were analyzed in the eight cantons of this province: Cayambe, Mejía, Pedro Moncayo, Pedro Vicente Maldonado, Puerto Quito, Rumiñahui, San Miguel de los Bancos, and Quito. These data revealed a higher number of infections in the canton of Quito. Consequently, the number of COVID-19 infections from March 2020 to March 2021 in Quito city was analyzed, along with the total deaths attributed to the virus during those two years. In this context, it was evident that national, provincial, and cantonal data were considered when selecting the study area, in order to corroborate the impact of atmospheric contaminants with the increase in COVID-19 cases in the city of Quito. This was one of the most affected cities nationwide, and it has a high concentration of air contaminants [37,62,63]. The descriptive statistics made it possible to summarize the results of the variables involved and recognize the possible patterns to which their causes were attributed.

#### 2.2.2. Atmospheric Contaminants vs. Covid-19 Infections in Quito City

**Figure 2:**
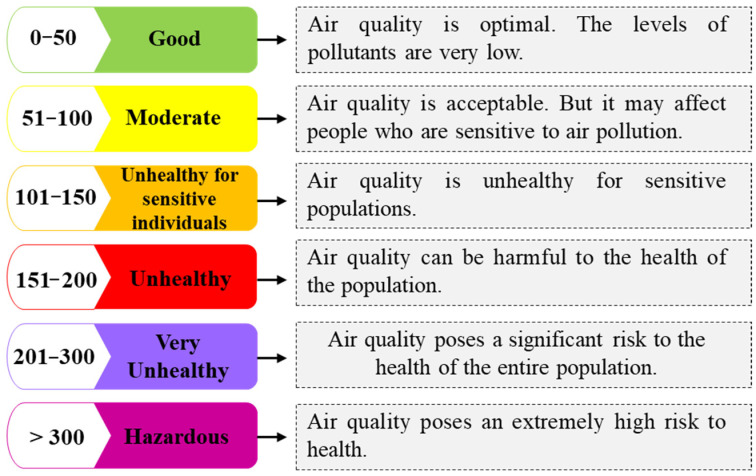
The Air Quality Index Scale. Adapted from Environmental Protection Agency, 2023.

A database of the atmospheric contaminants present in Quito city during March 2020 to March 2021 was built, such as CO, O_3_, SO_2_, PM_2.5_, and PM_10_. Based on the values obtained from two measurement stations in the city: the Historic Center and Belisario. The atmospheric contaminants with the greatest presence in the city were analyzed. The contaminants were measured based on the Air Quality Index (AQI) scale established by the United States Environmental Protection Agency (EPA) [64] (Figure 2). This scale has been adapted globally and may have local variations, but the basic principles are generally consistent.

These standards allowed air quality to be categorized into six levels, from “Good” to “Hazardous.” For this, atmospheric contaminants were valued based on the scale proposed by the Ministry of Environment, Water and Ecological Transition of Ecuador (MAATE) (Table 1). These values are presented in µg/m³ or mg/m³, depending on the type of contaminant. The data obtained were compared with the number of infections recorded in the same period to determine a graphical relationship. The Microsoft Excel statistical program was used for data analysis and the generation of tables and figures.

#### 2.2.3. Statistical Tests

A normality analysis was applied to the data obtained on COVID-19 infections, using the Statistical Package for the Social Sciences (SPSS 27.0^®^) software. In addition, the average values of each contaminant (O_3_, SO_2_, CO, PM_2.5_, PM_10_) were determined according to the Shapiro–Wilks and Kolmogorov–Smirnov tests [65,66]. A supervised learning approach was applied, using machine learning algorithms to model the influence of predictors on responses through regression analysis [67]. In a simple linear regression, each air contaminant was analyzed independently for its relationship with COVID-19 infections. In a mathematical model, a simple linear regression analyzes how a dependent variable, such as the number of COVID-19 infections, relates to an independent variable, such as the concentration of a contaminant in the air [68]. Five simple regression models were generated in RStudio 9.4.0.813654^®^. The statistical analysis allowed each model to be compared, with the purpose of correcting, debugging, and obtaining a final, solid model that demonstrates the relationship between atmospheric contaminants and COVID-19 cases. The five models analyzed the number of COVID-19 infections in relation to each atmospheric contaminant: O_3_, SO_2_, CO, PM_2.5_, and PM_10_ (one matrix for each). These models allowed us to verify those contaminants that were significantly related to the increase in COVID-19 cases. In addition, confounding factors were considered for the development of models, including various variables that had not been considered in the analysis, but that may have influencee the results. The confounding factors for the development of the models were temperature and precipitation.

## 3. Results

### 3.1. COVID-19 Infections

**Figure 3:**
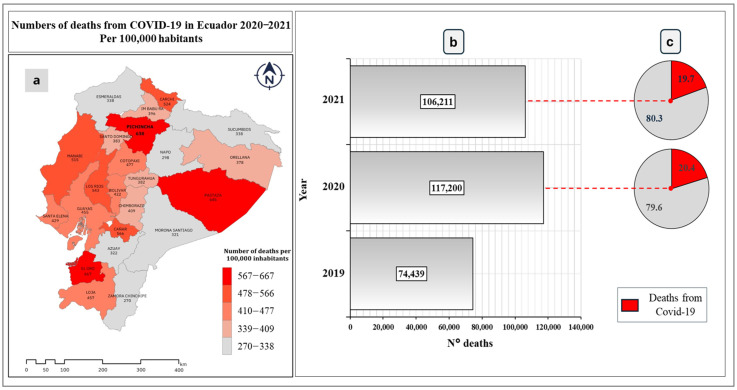
(**a**) Deaths from COVID-19 in Ecuador 2020–2021 per 100,000 inhabitants, (**b**) deaths in Ecuador from 2019 to 2021, (**c**) deaths caused by COVID-19 in 2020 and 2021.

Figure 3a,b show the deaths in Ecuador from 2019 to 2021. In 2019, 74,439 deaths were registered, a figure that increased by 57.4% for the year 2020, with a total of 117,200 deaths. A growth is shown in the number of deaths during the year 2020 compared to the base year. This increase can be attributed to the spread of the COVID-19 virus worldwide. However, for the year 2021, this figure decreased by 9.4%, with a total of 106,211 deaths. This can be attributed to the control measures taken to control the spread of the virus, such as social distancing, the mandatory use of masks, and vaccination campaigns. Furthermore, another factor that could have been influenced was the natural immunity that the population acquired after having contracted the virus and recovered. 

Figure 3c shows the deaths caused by COVID-19 in the country during 2020 and 2021. It shows a negative impact of the disease in mortality, with 20.4% of deaths being caused in 2020. That is, 23,921 people died from infection with the virus. This can be attributed to factors such as: (1) the saturation of health systems in the initial stage of the pandemic; (2) the lack of an effective cure; (3) the influence of psychological elements on the ability of the immune system to combat this disease; (4) exposure to atmospheric contaminants. On the other hand, in 2021, deaths from COVID-19 represented 19.7%, that is, 20,900 deaths from the virus. This 12.7% decrease in deaths compared to 2020 can be attributed to factors such as mass vaccination, social distancing, the body’s resilience to the virus, and the improvement of air quality during confinement due to the temporary reduction in levels of atmospheric contaminants.

**Figure 4:**
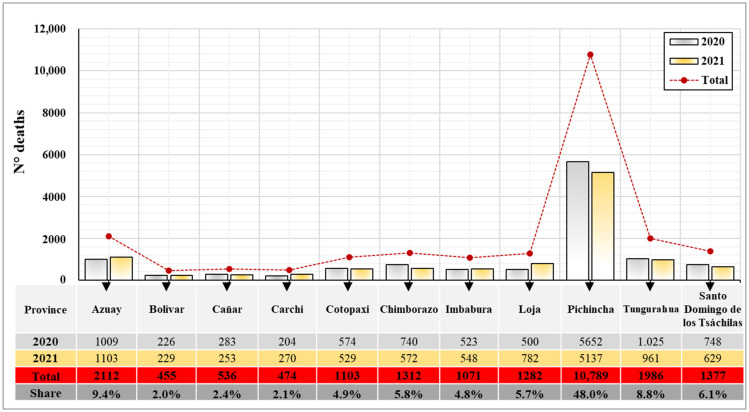
Deaths in the Andean natural region provinces caused by COVID-19 in 2020 and 2021.

The COVID-19 contagion in the Andean natural region has presented different dynamics over time. Figure 4 shows the deaths caused by this virus in 2020 and 2021 in relation to the provinces of this region. In general, it is evident that the highest percentage of deaths due to contagion is in the province of Pichincha with 48%, which represents 10,789 deceased people. In addition, it was identified that the provinces of Bolívar and Carchi presented a lower number of deaths due to infection with the virus, with 2% (455) and 2.1% (474), respectively. A significant difference is noted in the province of Pichincha in relation to the other provinces of the Andean natural region, possibly marked by the demographic composition, considering that this province has a higher population density. This factor, combined with the constant mobility of people inside and outside the province, caused the rapid spread of the virus.

**Figure 5:**
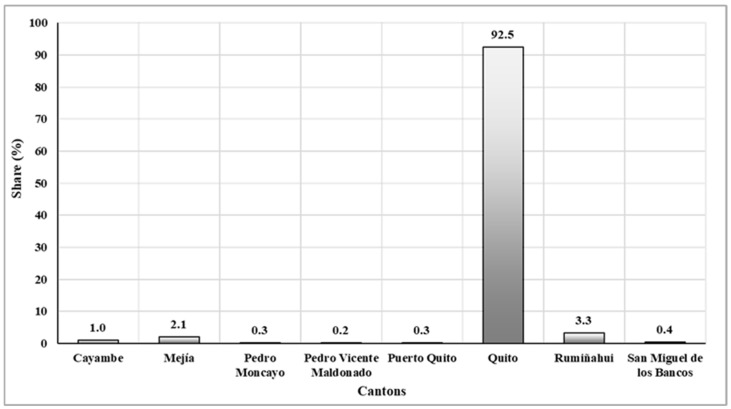
COVID-19 infections in the province of Pichincha cantons from March 2020 to December 2021.

Due to the negative impact of COVID-19 in the province of Pichincha, it was considered important to analyze infections due to this virus in the cantons of the province from March 2020 to December 2021 (Figure 5). During this period, it was determined that Quito had been one of the cantons most affected by the pandemic. It reached the highest percentage of 92.5% in relation to the other cantons, where the percentages remained consistently low, oscillating between 0.2% and 3.3%.

**Figure 6:**
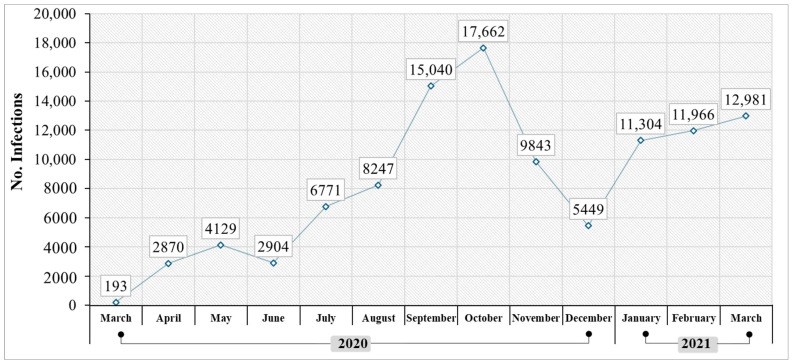
Monthly COVID-19 infections in Quito city during March 2020 to March 2021.

It is important to consider that COVID-19 infections in the Quito canton were not distributed homogeneously, with a greater presence of contagion in places with a high concentration of people, such as the urban area. Figure 6 shows the monthly infections in Quito city from March 2020 to March 2021. In this period, there were a total of 109,359 infections. In the first months of the pandemic, significant peaks in contagion cases were observed due to the rapid spread of the virus and the lack of effective control measures, with an increasing trend from March to October 2020. 193 infected people were registered in March, while in October, there was an increase of 90.5% in seven months, reaching a total of 17,662 infected people. Subsequently, in December 2020, a reduction in the number of infections was recorded, ending the year with 5449 cases. This can be attributed to the implementation of protective measures, social distancing, and reduced mobility in the city. However, by 2021, infections had increased again, reaching 12,981 cases by March of that year. This fact can be attributed to the appearance of new variants of the virus, such as Delta and Omicron, and the reduction in compliance with preventive and control measures [69,70].

**Figure 7:**
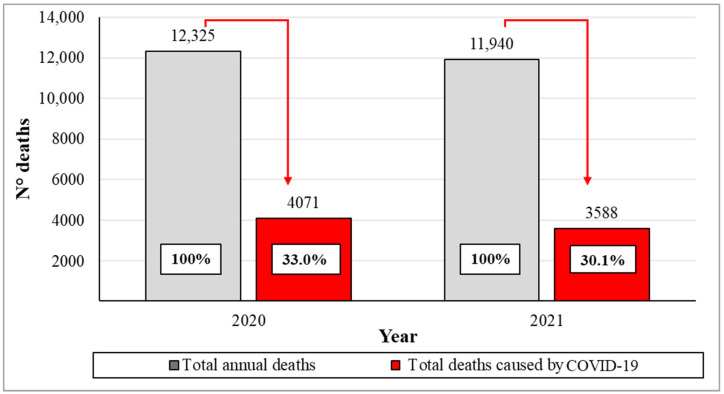
Total deaths and deaths caused by COVID-19 in the city of Quito during the year 2020 and 2021.

COVID-19 directly and indirectly influenced the increase in population mortality. Figure 7 shows the total deaths and those caused by the virus in Quito city during 2020 and 2021. In 2020, a total of 12,325 deaths were recorded, of which 33% died due to the contagion. In 2021, a total of 11,940 deaths were reported, with 30.1% of them related to the virus. The analysis of factors that contributed to increasing the magnitude of the impact of COVID-19 on human health in Quito became an important aspect, considering that the virus was the cause of a third of the deaths registered in 2020 and 2021.

In this context, the increase in infections and deaths can be associated with various factors. Quito, as the country’s capital and the second largest city nationwide, has a high population density and a continuous flow of people, which allowed the spread of the virus and the saturation of the health systems. Furthermore, being a point of economic, commercial, and tourist interest at the national level, it became a focal point for the rapid spread of the virus. Besides, at an environmental level, it is important to consider the quality of the city’s air, particularly the presence of atmospheric contaminants that influence people’s health, mainly those who suffer from respiratory conditions.

### 3.2. Atmospheric Contaminants vs. COVID-19 Cases in Quito City

**Figure 8:**
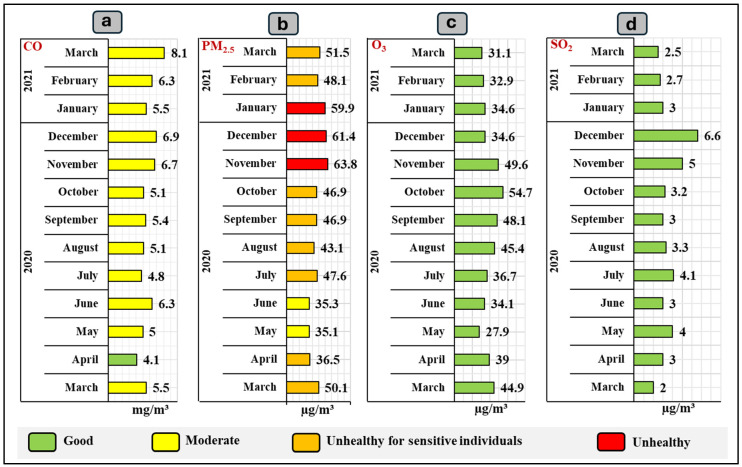
Concentration of atmospheric contaminants in Quito city during the period March 2020 to March 2021 obtained from the Historic Center station: (**a**) CO; (**b**) PM_2.5_; (**c**) O_3_; (d) SO_2_.

Figure 8 indicates the concentration of atmospheric contaminants Quito city recorded by the Historic Center station from March 2020 to March 2021. CO levels range from 4.1 mg/m³ to 8.1 mg/m³ (Figure 8a). Most of these values are within the “Moderate” range (4.5–9.0 mg/m³), except for the month of April, which, at 4.1 mg/m³, is below the threshold of 4.4 mg/m³ for the “Good” category. PM_2.5_ levels range from 35.1 to 63.8 µg/m³ (Figure 8b). Most values are in the “Unhealthy for Sensitive Individuals” category, except for those in November (63.8 µg/m³), December (61.4 µg/m³) and January (59.9 µg/m³), which are in the “Unhealthy” category. O_3_ concentrations, on the other hand, ranged between 27.9 and 54.7 µg/m³ (Figure 8c). All values are in the “Good” category. SO_2_ concentrations also ranged between 2.0 and 6.6 µg/m³ (Figure 8d), which are below the 20 µg/m³ threshold and are therefore in the “Good” category.

**Figure 9:**
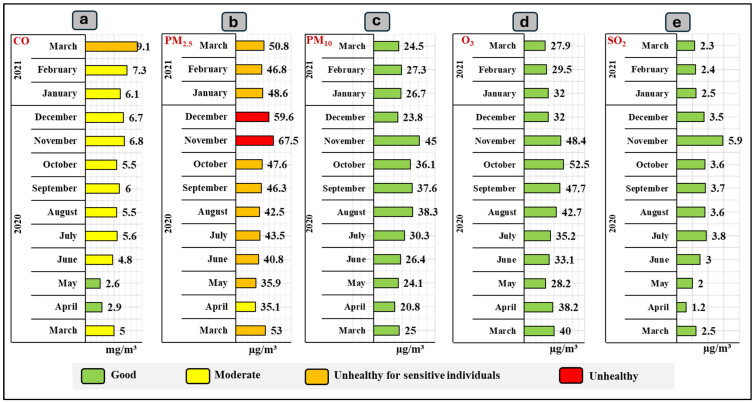
Concentration of atmospheric contaminants in Quito city during the period March 2020 to March 2021 obtained from the Belisario station: (**a**) CO; (**b**) PM_2.5_; (**c**) PM_10_; (**d**) O_3_; (**e**) SO_2_.

Figure 9 indicates the concentration of atmospheric contaminants in Quito city recorded by the Belisario station from March 2020 to March 2021. CO values remained relatively stable, ranging between 2.6 mg/m³ and 9.1 mg/m³ (Figure 9a). A gradual increase was observed from May 2020 until reaching the maximum in March 2021. CO levels remained mostly within the “Moderate” category with values ranging between 4.8 and 7.3 mg/m³. On the other hand, PM_2.5_ levels were mostly classified as “Unhealthy for Sensitive Individuals”, with values between 36.5 and 63.8 µg/m³ (Figure 9b). However, in November and December 2020, concentrations of 67.5 and 59.6 µg/m³ were recorded respectively, which corresponds to the “Unhealthy” category. The concentrations of coarse particulate matter (PM_10_) were also analyzed (Figure 9c). These values fluctuated between 20.8 and 45 µg/m³, which are within the “Good” category. As with PM_2.5_, a peak was recorded in November 2020, followed by a reduction in the following months. Regarding O_3_ levels (Figure 9d), there is a variation between 27.9 and 52.5 µg/m³. All values are within the “Good” category. Finally, regarding SO_2_ values (Figure 9e), low levels of this contaminant were observed. These values remained in the “Good” category throughout the period, with 1.2 and 5.9 µg/m³.

**Figure 10:**
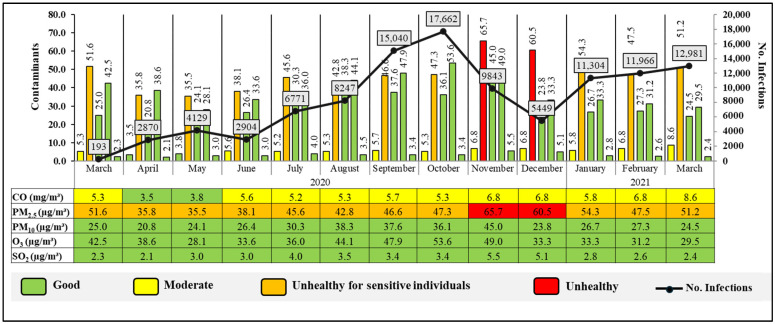
Concentration of atmospheric contaminants and COVID-19 infections in Quito city during the period March 2020 to March 2021.

Figure 10 shows the relationship between the concentrations of atmospheric contaminants (CO, O_3_, PM_2.5_, SO_2_, and PM_10_) with the number of COVID-19 cases. In March 2020, most contaminants were found at moderate levels, except for PM_2.5_ with 51.6 µg/m³, which is at an “Unhealthy for sensitive individuals” level. COVID-19 infections were low, with 193 in total. On the other hand, from June to September, an increase is observed for all contaminants, with levels fluctuating between “Moderate”, “Unhealthy for sensitive individuals”, and “Unhealthy”. During this period, infections increased significantly, reaching a peak in September. For example, the data indicate that in October 2020, when there was the highest number of infections, with 17,662 infected people, there were also high levels of CO and PM_2.5_, with 5.3 mg/m^3^ and 47.3 µg/m^3^, respectively. Besides, in this month, ozone levels (O_3_), despite being within the appropriate ranges, also show an increase, reaching their maximum point at 53.6 µg/m³. On the other hand, a significant increase in PM_2.5_ is also indicated in November 2020 (65.7 µg/m³), coinciding with a high number of infections (9843). 

These findings suggest a possible relationship between the concentration of air contaminants and the incidence of COVID-19 infections in Quito. The months with the highest concentrations of these contaminants coincided with the increase in infections, which could indicate that poor air quality contributes to the spread of the virus or aggravates the disease in infected individuals. It is important to consider that the presence of air contaminants in the city of Quito may be influenced by factors such as: (1) location, (2) development of industrial activities, (3) greater number of vehicles, and (4) increase in the vehicle fleet. The flow of water is very fluid. Its location in a valley surrounded by mountains facilitates the accumulation of air contaminants. In addition, the presence of industrial and commercial activities in factories and production plants also contributes to the emission of gases and particles. The high number of vehicles is one of the main sources of air pollution, mainly due to emissions from mobile sources and the generation of greenhouse gases such as CO_2_, NOx, and suspended particles [71,72].

### 3.3. Statistical Tests

**Figure 11:**
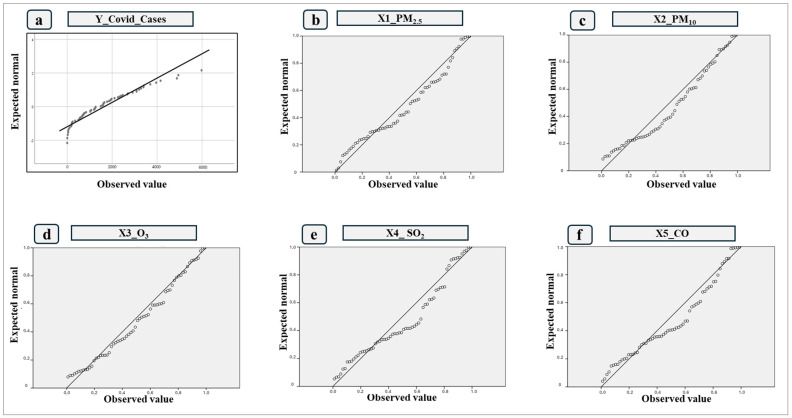
Normality tests. (a) Y_Covid_cases; (b) X1__PM2.5_; (c) X2_PM_10_; (d) X3_O_3_; (e) X4_SO_2_; (f) X5_CO.

Table 2 and Figure 11 show a normality analysis of daily COVID-19 infections and the average of the values of each contaminant (PM_2.5_, PM_10_, O_3,_ SO_2_, and CO). The results of these tests provided important information about the way the data is distributed.

There are significance values less than 0.05 in the case of the Y variables, as for X3_SO_2_ (Figure 11e), which shows that the data do not follow a normal distribution. On the other hand, in the case of the variables X1_PM_2.5_ (Figure 11b), X2_PM_10_ (Figure 11c), X3_O_3_ (Figure 11d), and X5_CO (Figure 11f), a significance level greater than 0.05 was obtained, showing a normal distribution.

Five mathematical models were developed, one for each atmospheric contaminant (PM_2.5_, PM_10_, O_3_, SO_2_zo, and CO) that reflected the incidence of these with the increase in cases due to COVID-19 infection from March 2020 to March 2021 (Table 3).

**Figure 12:**
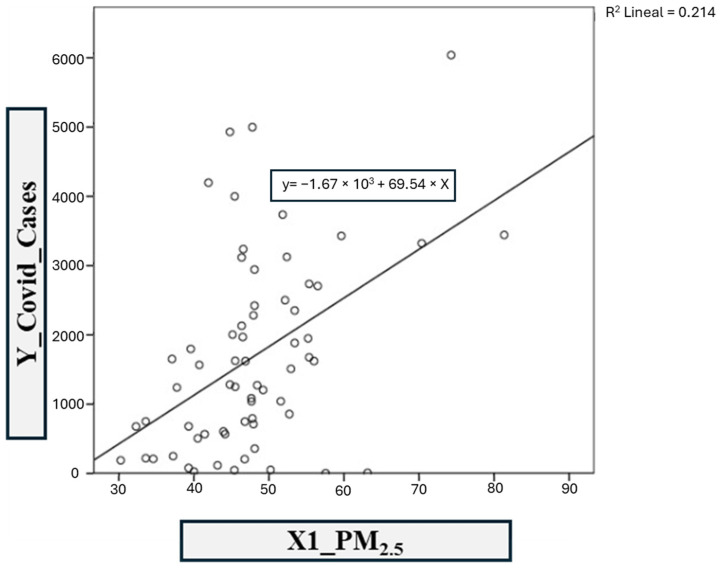
Model 1: COVID-19 vs. PM_2.5._

The first model was based on COVID-19 infections vs. the PM_2.5_ contaminant (Figure 12). The linear regression coefficients *R*^2^ and adjusted *R*^2^ obtained in SPSS are 0.214 and 0.201, respectively. This shows a moderate correlation between PM_2.5_ concentrations and cases of contagion. Therefore, model 1 is defined with the following equation:

y = −1667.48x − 69.54(1)

**Figure 13:**
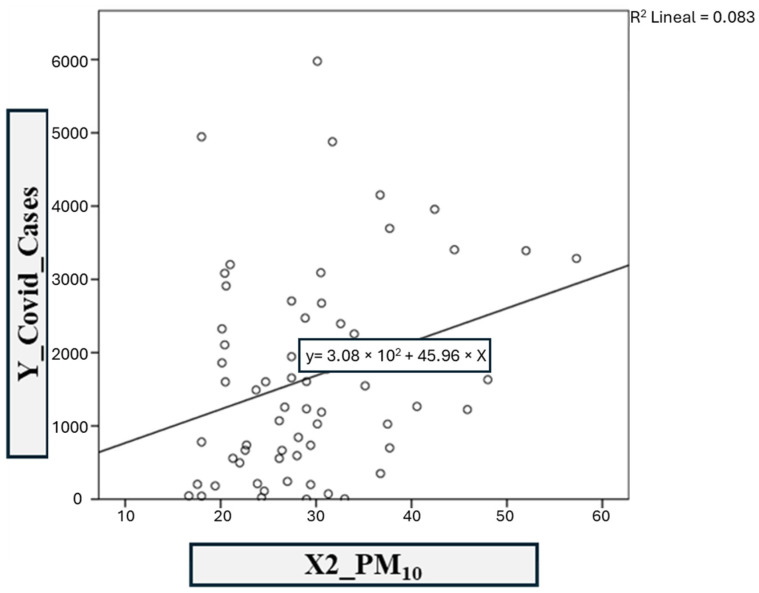
Model 2: COVID-19 vs. PM_10._

The second model was based on COVID-19 infections versus the PM_10_ contaminant (Figure 13). The linear regression coefficients *R*^2^ and adjusted *R*^2^ obtained in SPSS are 0.083 and 0.068, respectively. This correlation is considered scarce. Therefore, model 2 is defined with the following equation:

y = 308.36x − 45.96(2)

**Figure 14:**
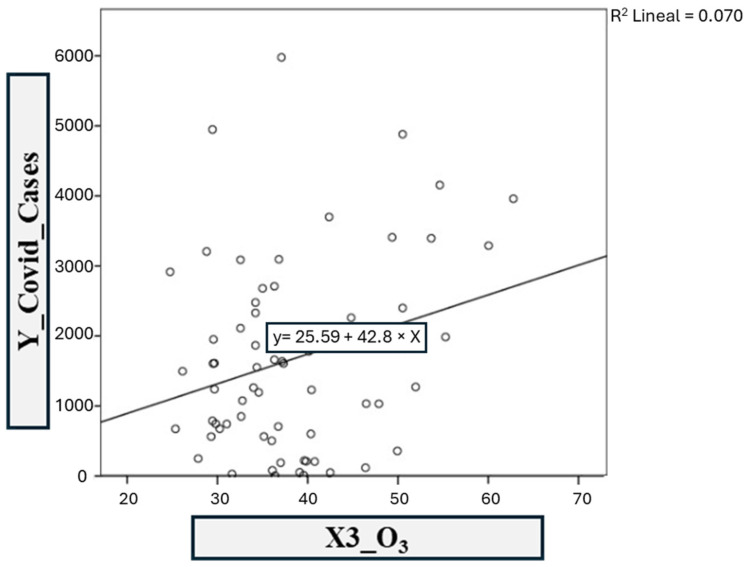
Model 3: COVID-19 vs. O_3._

The third model was based on COVID-19 infections vs. the O_3_ contaminant (Figure 14). The linear regression coefficients *R*^2^ and adjusted *R*^2^ obtained in SPSS are 0.070 and 0.055, respectively. This shows a moderate correlation between the atmospheric contaminant SO_2_ and COVID-19 infections. Therefore, model 3 is defined as:

y = 25.590x − 42.80(3)

**Figure 15:**
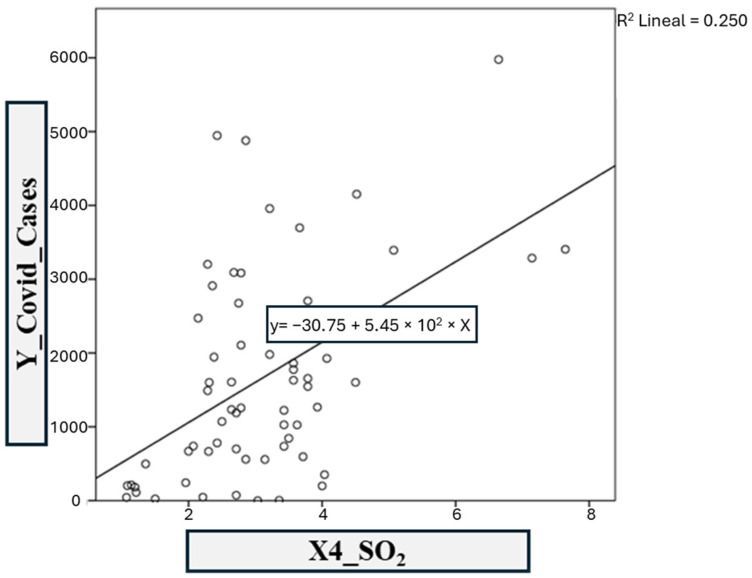
Model 4: COVID-19 vs. SO_2._

The fourth model was based on COVID-19 infections vs. the SO_2_ contaminant (Figure 15). The linear regression coefficients *R*^2^ and adjusted *R*^2^ obtained in SPSS are 0.250 and 0.238, respectively. This shows a moderate correlation in model 4, defined by the following equation:

y = −30.75 – 544.95(4)

**Figure 16:**
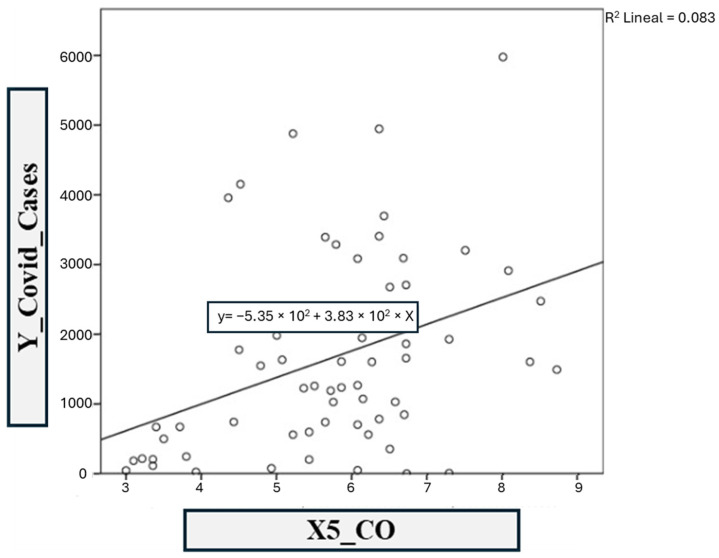
Model 5: COVID-19 vs. CO.

Finally, the fifth model was based on COVID-19 infections vs. the CO contaminant (Figure 16). The linear regression coefficients *R*^2^ and adjusted *R*^2^ obtained in SPSS are 0.143 and 0.129, respectively. This shows a moderate correlation in model 5, defined by the following equation:

y = −535.292x − 382.915(5)

The models developed in this study show different levels of correlation between contaminants and the increase in COVID-19 cases. The fourth model, associated with sulfur dioxide (SO_2_), shows the significant correlation between the analyzed contaminants and COVID-19 cases, with a coefficient of determination (*R²*) of 0.250. This finding shows that SO_2_ influenced the dynamics of the pandemic. Exposure to SO_2_ has been associated with adverse effects on respiratory health, which could increase vulnerability to viral infections such as COVID-19. On the other hand, the first PM_2.5_ model presents an *R²* of 0.214, indicating a moderate correlation. This suggests that as PM_2.5_ levels increase, so do COVID-19 cases. Scientific literature has documented that exposure to PM_2.5_ can weaken the immune system and increase susceptibility to respiratory infections, which could explain this correlation. Ozone (O_3_) also shows a moderate correlation (*R²* = 0.070), indicating that elevated ozone levels may be associated with an increase in COVID-19 cases. Ozone exposure has been linked to lung inflammation and exacerbation of respiratory diseases, which could contribute to greater severity of COVID-19 in exposed populations. Carbon monoxide (CO) is another contaminant that shows a moderate correlation (*R²* = 0.148), indicating that there is a relationship between CO levels and the increase in COVID-19 cases, although this relationship is less pronounced than that observed with other contaminants. Finally, the model analyzing the relationship between PM_10_ and COVID-19 cases shows a low correlation (*R²* = 0.083). This suggests that although PM_10_ is a relevant contaminant, its direct impact on the increase in COVID-19 cases may be less significant compared to other contaminants. However, this does not diminish the importance of monitoring PM_10_ levels, as it remains a risk factor for respiratory health.

In this context, it is evident that the model with the highest incidence in COVID-19 infections is the SO_2_ model 4. On the other hand, the PM_2.5_, O_3_, and CO models show moderate correlations, suggesting that these contaminants may have some effect on infections, although they are not as significant as SO_2_. The PM_10_ model 2 shows very low correlations, indicating that this contaminant has little impact on COVID-19 infections.

**Figure 17:**
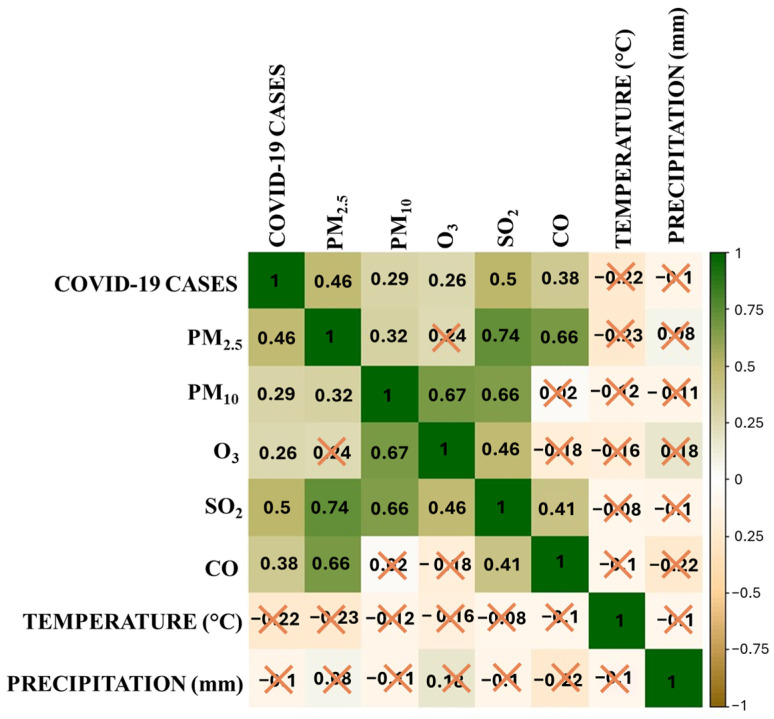
Correlation analysis.

Figure 17 shows the correlation of COVID-19 infections with atmospheric contaminants and environmental variables (temperature and precipitation). Moderate positive correlations are evident with PM_2.5_ (0.46), SO_2_ (0.50), and CO (0.38), suggesting that increases in these contaminants are associated with a rise in infections. In terms of environmental variables, temperature and precipitation show low (negative) correlations with COVID-19 cases, indicating that they do not have a significant direct relationship with the increase in infections. Environmental variables, such as temperature and precipitation, could modify the impact of contaminants on infections; however, since they do not have significant correlations with COVID-19 cases, their effect in this relationship is minimal. Because they are not correlated with COVID-19 cases, environmental variables may act as confounding factors, meaning they can influence the relationship between contaminants and infections without being direct determinants of the number of cases.

**Figure 18:**
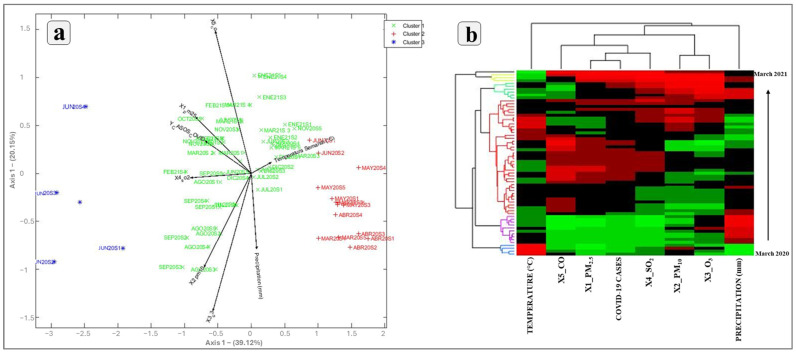
Statistical analyses: (**a**) multivariate analysis Hj-biplot; (**b**) biclustering analysis.

Figure 18 shows the statistical analyses of COVID-19 infections with contaminants and environmental variables (temperature and precipitation). Three clusters are displayed: Cluster 1 (green), Cluster 2 (red), and Cluster 3 (blue) (Figure 18a). Clusters 1 and 2 group the weeks of the months in which contamination and infection levels were moderate to high, such as September, October, November, and December 2020. Weeks with high contamination levels tend to cluster with higher numbers of COVID-19 infections, reinforcing the positive correlation observed between some pollutants (PM_2.5_, SO_2_, and CO). Meanwhile, Cluster 3 shows weeks with atypical or significantly different behavior compared to the others, possibly due to a low correlation with precipitation, temperature, and contamination variables. Additionally, the angle of the temperature vector is small relative to the infection vector, indicating a negative relationship between temperature and infections, although it is not as strong as the relationship observed with contamination (such as PM_2.5_ or PM_10_).

On the other hand, Figure 18b shows a biclustering analysis through a heatmap that integrates COVID-19 infection data and their relationship with atmospheric contaminants and environmental factors (Figure 18b). In several weeks of October 2020, high levels of PM2.5 coincide with high rates of COVID-19 infections, indicating a correlation between the levels of certain air contaminants. Similarly, in several weeks where carbon monoxide (CO) and sulfur dioxide (SO_2_) levels are high (red), COVID-19 infections also show an increase. This behavior reinforces the idea that air pollution is related to an increase in COVID-19 cases, possibly by increasing the vulnerability of the respiratory system. In contrast, during weeks when temperatures are lower (green colors in the temperature column), infection levels tend to be high (red colors), indicating a negative correlation between temperature and infections. Additionally, precipitation does not show a correlation with the increase in infections, as it does not have a significant direct relationship with COVID-19 cases. The weeks that cluster in nearby branches in the dendrogram tend to share similar values in contamination variables and infection cases. This may indicate seasonal trends or specific time periods where air conditions and infections evolved together.

## 4. Discussion

The relationship between air quality and public health has become critically important in the context of the COVID-19 pandemic. The analysis of COVID-19 cases has revealed a dynamic evolution in time and space [73]. In the findings of this study, periods of acceleration in the transmission of the virus are observed, followed by stabilization or a decrease in the number of cases. Since its appearance, several scientific studies have focused on studying the behavior of these infections [11,16,17], the transmission patterns [74] and the factors that contribute to the spread of the virus [75]. Among these factors, air quality and the presence of atmospheric contaminants have emerged as areas of scientific interest [29,43,55,56]. The consequences of air quality degradation are manifested in a significant percentage of global mortality each year [76]. Therefore, in recent years, the impact on people’s health and its consequences over time has been the subject of several studies [77,78]. The evolution of infections has not been uniform, but has depended on various factors. The authors believe that this highlights the importance of analyzing air quality as a factor that affects the health of people infected by the virus.

Globally, it was found that contaminant emissions such as particulate matter (PM) [27], carbon monoxide (CO), carbon dioxide (CO_2_) [25], sulfur oxides (SOx), and nitrogen oxides (NOx) [79] are generated in greater quantities [80], particularly in urban areas [81]. This contrasts with the data taken at the stations in the study area for this research, which evidences the accumulation of these contaminants in Quito, particularly CO, O_3_, SO_2_, PM_2.5_, and PM_10_. The concentration of these contaminants can be influenced by industrial activity, vehicular traffic, and climatic conditions [29], which act as vectors of virus transmission, facilitating its spread in densely populated areas [46]. Previous research shows that atmospheric contaminants compromise the immune system, causing various health problems [82,83,84]. For instance, they have influenced the increase in cases of hospitalization for respiratory diseases [29,62,63], the development of cardiovascular diseases [85], cancer [86], and complications during pregnancy [87]. Furthermore, in the context of the COVID-19 pandemic, a possible relationship has been found between exposure to air contaminants and the increase in cases or the severity of the disease [30,31,73]. This may be because air contaminants can damage the lungs and respiratory system, making people more vulnerable to complications caused by the virus [16]. We believe that people who live or work in urban areas are exposed to higher levels of air pollution, which increases their risk of developing respiratory-related health problems, such as COVID-19.

It is important to understand the impact of air contaminants on the spread of this disease in urban environments. Understanding this relationship is complex and multifaceted [46]. According to some studies, in urban environments with high levels of air pollution, COVID-19 can be transmitted faster than in rural areas [44,67,73]. Based on this, it is important to highlight that these studies support the findings in the study area, where peaks of contagion were identified during the months in which there was a greater presence of atmospheric contaminants (October and September 2020). Furthermore, this relationship was analyzed through mathematical modeling. This type of model can be used to investigate the association between the incidence of atmospheric contaminant concentrations and various diseases [88,89]. Mathematical models can be used to quantify the strength and direction of this relationship [57]. In this study, the mathematical modeling developed provided a powerful framework to explore and quantify the relationships between complex and dynamic variables. These variables provided information to better understand the dynamics of the pandemic. Quantitative variables, such as the number of COVID-19 infections and the concentration of atmospheric contaminants, offered specific data on the incidence of contaminants in the virus [90]. We believe that, while models are certainly useful tools for understanding the dynamics of the pandemic, they should be used with caution and in combination with other types of evidence and expert knowledge.

Furthermore, the final mathematical model showed a higher incidence of COVID-19 infections with contaminants such as CO_2_, O_3_, and SO_2_. This can be associated with studies suggesting that exposure to air contaminants influences the increase in COVID-19 cases [26,27]. For instance, in China, an incidence of contaminants such as CO, NO_2_, O_3_, PM_2.5_, and PM_10_ has been revealed [91]. In Europe, prolonged exposure to high concentrations of NO_2_ and SO_2_ may contribute to COVID-19 mortality rates [92]. In this regard, the main finding of this research is the validity of the mathematical model, supported by the use of linear regression techniques [93] to identify significant correlations, for analyzing the relationship between a dependent variable [94], in this case COVID-19 infections, and one or more independent variables [90], in this case air contaminants. We believe that mathematical models can contribute to obtaining data that can help create policies and measures to mitigate the effects of the pandemic and improve air quality. However, it is important to keep in mind that these models are simplifications of reality and are subject to uncertainty, so additional research is needed to fully understand this relationship.

However, it is important to highlight the limiting factors when analyzing the relationship between air contaminants and COVID-19. One of these was the limitation and reliability of data. In several studies, infectious disease models depend on accurate and reliable data for validation [95]. Data on COVID-19 and air quality may be incomplete or biased [96], especially in areas with less developed epidemiological and environmental surveillance systems [97], as is the case with the Ecuadorian health system. This can introduce uncertainty into the model’s results and limit its usefulness for decision-making [98,99]. Another factor to consider is geographic and temporal variability. Mathematical models may have difficulty capturing this variability adequately, which limits their ability to generalize the results to different geographical and temporal contexts [100]. A third factor is associated with limitations in causality. Association models between COVID-19 and air quality can identify statistically significant correlations, but establishing a strong causal relationship requires additional evidence, such as longitudinal studies, controlled experiments, or analysis of underlying biological mechanisms [101]. Finally, another limitation was the sensitivity to assumptions and parameters [102]. In this case, several models were developed to obtain the final model, with the changes that were considered necessary. However, changes in model structure or parameter values can lead to significantly different results [103]. We believe it is important to take these limitations into account when interpreting the results of COVID-19 studies analyzed through mathematical models and using them to inform public health policies and measures.

Globally, mathematical models are increasingly used to forecast the future of epidemics, such as COVID-19 [83]. These predictions have far-reaching consequences, as they provide relevant information so that governments can act quickly to stop or mitigate an epidemic [86,87]. Mathematical models can be useful tools for making public health decisions and ensuring the optimal use of resources [57]. This is intended to reduce morbidity and mortality associated with the COVID-19 pandemic, but only if the models are rigorously evaluated, and their projections are robust and reliable.

## 5. Conclusions

The significant increase in deaths between 2019 and 2020, followed by a decrease in 2021, reflects the direct impact of COVID-19 on national mortality rates. In the Andean natural region, the province of Pichincha, particularly Quito, experienced a disproportionate burden of COVID-19 deaths, with significant spikes in the first months due to the rapid spread of the virus associated with its high population density and subsequent declines attributed to the protection measures followed at the national level.

Predictions resulting from the analysis of the relationship between COVID-19 and air contaminants using statistical tests and mathematical models can help inform public health policies and mitigation measures to reduce the impact of the pandemic. In this sense, this study provides a comprehensive assessment of the relationship between COVID-19 infections and environmental contaminants in Quito, revealing significant findings on how air pollution levels can influence the spread and severity of infections. Our results indicate that air contaminants, such as SO_2_, O_3_, CO, and PM_2.5_, are associated with an increase in the incidence and severity of COVID-19 cases in the city. These findings underscore the need for close monitoring of air quality and public policies aimed at reducing pollution to mitigate adverse impacts on respiratory health, especially in the context of emerging pandemics. 

The research therefore contributes to the understanding of how specific environmental factors can interact with pathogens, providing a scientific basis for the implementation of more effective public health strategies tailored to local conditions. In addition, these findings underscore the need to address both the pandemic and air pollution comprehensively. Furthermore, improving air quality could benefit public health in both the short and long term, reducing respiratory disease vulnerability and enhancing readiness for future pandemics.

## Figures and Tables

**Table 1:** Scale for assessing air contaminants.

Category	AQI	Air Contaminants				
		PM_10_ (µg/m³)	PM_2.5_ (µg/m³)	O_3_ (µg/m³)	SO_2_ (µg/m³)	CO (mg/m³)
Good	0–50	≤54	≤12.0	≤180	≤20	≤4.4
Moderate	51–100	55–154	12.1–35.4	181–240	21–50	4.5–9.0
Unhealthy for Sensitive individuals	101–150	155–254	35.5–55.4	241–300	51–150	9.1–15.0
Unhealthy	151–200	255–354	55.5–150.4	301–400	151–200	>15.0
Very Unhealthy	201–300	355–424	150.5–250.4	400–500	201–300	-
Hazardous	>300	>424	>250.4	>500	>300	-

**Table 2:** Data normality analysis.

	Kolmogorov–Smirnov ^a^			Shapiro–Wilk		
	Statistic	gl	Sig.	Statistic	Gl	Sig.
Y_Covid_Cases	0.107	64	0.101	0.9412	64	0.1854
X1_PM_2.5_	0.064	64	0.057	0.9734	64	0.0789
X2_PM_10_	0.065	64	0.605	0.9784	64	0.0893
X3_O_3_	0.073	64	0.401	0.9862	64	0.0623
X4_SO_2_	0.1351	64	0.005	0.9471	64	0.0002
X5_CO	0.0732	64	0.446	0.9804	64	0.1086

^a^ Lilliefors significance correction.

**Table 3:** Simple linear regression models based on COVID-19 infections in relation to air contaminants.

SPSS				
Model	R	R Squared	R Squared Adjusted	Error
X1_PM_2.5_	0.463 ^a^	0.214	0.201	1235.42
X2_PM_10_	0.288 ^a^	0.083	0.068	1334.69
X3_O_3_	0.264 ^a^	0.070	0.055	1343.99
X4_SO_2_	0.500 ^a^	0.250	0.238	1207.10
X5_CO	0.378 ^a^	0.148	0.129	1290.06

^a^ Predictors (constant).

## Data Availability

All the data generated and analyzed during this study are included in this published article.
